# Research Progress of Drug Delivery Systems Consisting of Hydrogels Loaded with Extracellular Vesicles in Tumor Therapy

**DOI:** 10.32604/or.2025.067586

**Published:** 2025-11-27

**Authors:** Shaojian Zou, Lipeng Zhang, Xiang Chen, Zhuomin Wang, Xinhui Zhu, Dandong Luo, Shengxun Mao, Zhen Zong

**Affiliations:** 1 Huan Kui Academy, Jiangxi Medical College, Nanchang University, Nanchang, 330006, China; 2 Department of Gastrointestinal Surgery, The Second Affiliated Hospital, Jiangxi Medical College, Nanchang University, Nanchang, 330006, China; 3 The First Clinic Medical College, School of Medicine, Nanchang University, Nanchang, 330006, China; 4 Department of Gastrointestinal Surgery, Department of General Surgery, The Sixth Affiliated Hospital, Sun Yat-sen University, Guangzhou, 510655, China

**Keywords:** Hydrogels, extracellular vesicles (EVs), drug delivery systems, tumor therapy, passive targeting

## Abstract

Traditional cancer therapies have limitations like poor efficacy on advanced tumors, healthy tissue damage, side effects, and drug resistance, creating an urgent need for new strategies. Hydrogels have good biocompatibility and controlled release, while extracellular vesicles (EVs) enable targeting and bioactive transport. This review systematically summarizes hydrogels and EVs, focusing on the construction of hydrogel-EV delivery system, key influencing factors, drug delivery mechanisms, and tumor therapy apps, clarifying their synergies. The system overcomes single-carrier flaws, construction methods/key factors affect performance, preclinical studies have confirmed efficacy in multiple therapies, but large-scale production and *in vivo* stability challenges remain, yet it promises to overcome the limits of traditional therapy for precision oncology.

## 1 Introduction

Cancer is a leading cause of morbidity and mortality worldwide [1]. Developing effective cancer treatments remains a central focus of medical research. Conventional approaches—surgery, radiation therapy, and chemotherapy—remain cornerstones of cancer treatment. However, these methods have significant limitations. Surgical resection is effective for localized, early-stage tumors but is often ineffective against advanced cancers, especially those with metastasis [2]. While radiation therapy can destroy cancer cells, it also causes significant damage to surrounding healthy tissues [3]. This leads to a range of side effects. Chemotherapy’s nonspecific action targets both cancerous and healthy cells, resulting in serious side effects, including alopecia, nausea, vomiting, and immune deficiencies. These side effects significantly impact quality of life and treatment tolerance [2].

In recent decades, advances in molecular biology and targeted therapies have led to the development of targeted drugs and immunotherapies, offering new hope for tumor treatment. But tumor cells may develop resistance with prolonged treatment [4]. Cells may evade drug-induced cell death through various mechanisms, regardless of whether the treatment is chemotherapy, targeted therapy, or immunotherapy. These mechanisms include upregulation of drug transporters that facilitate drug efflux, alterations in drug-metabolizing enzymes leading to inactivation, compensatory activation of downstream pathways that bypass the drug’s target, and the presence of cancer stem cells [5]. These resistance mechanisms diminish the efficacy of previously effective treatments, highlighting the urgent need for innovative therapeutic strategies. A promising solution lies in the advancement of drug delivery: the combination of hydrogels and EVs has shown great potential, making it a central focus of the current approach may overcome these limitations and pave the way for more effective and targeted cancer therapies.

Hydrogels, with their three-dimensional network structure, are widely used in biomedical fields such as tissue engineering, wound healing, and drug delivery due to their excellent biocompatibility, tunable properties, and ability to load and release drugs in a controlled way [6–8]. This intelligent release mechanism enables on-demand drug delivery, tailored to the tumor’s pathological characteristics, thereby enhancing therapeutic efficacy and minimizing drug waste and systemic toxicity.

Extracellular vesicles, including exosomes and microvesicles, are key mediators of intercellular communication, which naturally target specific cells or tissues to deliver therapeutic agents in a cell-specific manner [9]. These vesicles transport bioactive molecules, such as proteins and nucleic acids (e.g., miRNA and mRNA), which are involved in essential physiological and pathological processes, including cell signaling and immune regulation [10].

The drug delivery systems consisting of hydrogels loaded with extracellular vesicles offer several unique advantages in tumor therapy, providing new hope for cancer treatment (Table 1). Hydrogels serve as efficient carriers, protecting EVs and their anti-tumor payloads from premature degradation or clearance *in vivo*, thereby ensuring stable drug delivery to the tumor site. Their three-dimensional network structures provide physical and chemical protection by embedding the extracellular vesicles and drugs. Additionally, the hydrogels’ excellent biocompatibility prevents excessive immune recognition and attack, minimizing the risk of adverse effects; another advantage lies in the superior targeting ability of the extracellular cells secrete various specific signaling molecules during growth and natural homing properties of EVs enable them to recognize these molecules and specifically target tumor tissues [11], increasing drug concentration at the tumor site while minimizing toxicity and side effects in healthy tissues [12]. Preclinical studies of various solid tumors have demonstrated the significant anti-tumor effects of cell membrane vesicle-loaded hydrogel drug delivery systems [13,14].

**Table 1:** A comparative analysis table of the key research contents of extracellular vesicles carrying hydrogel systems at        present

Hydrogel type	Extracellular vesicle types	Loaded with drugs/ active ingredients	Core achievements	References
PLGA-PEG-PLGA hydrogel	Exosomes	Vascular endothelial growth factor	The programmable release of vascular endothelial growth factor (VEGF) and exosomes has been shown to enhance bone regeneration. Compared to conventional separate administration, combined delivery with spatiotemporal release more effectively harnesses synergistic effects enhances therapeutic efficacy, and minimizes risks associated with frequent dosing.	[15]
GelMA hydrogel	Exosomes	Adriamycin	A high degree of methacrylation enhances hydrogel stiffness, enables stable encapsulation of extracellular vesicles (EVs), and facilitates sustained drug release, thereby effectively inhibiting tumor cell migration and aggregation. Compared to traditional chemotherapy, this approach reduces dosing frequency by mitigating rapid drug clearance. Moreover, EVs derived from citrus fruits, including lemon, exhibit low immunogenicity and help minimize allergic responses.	[16]
Degradable hydrogel	Exosomes	BCG vaccine	Enables site-specific and sustained release of BCG at tumor locations, thereby activating anti-tumor immune responses. Compared to systemic BCG administration, this localized approach minimizes adverse effects such as fever and fatigue associated with systemic immune activation, while local accumulation improves antigen presentation efficiency.	[17]
Gelatin methacryloyl hydrogel	Exosomes	VH298	The bioactive components within extracellular vesicles (EVs)promote cell proliferation and migration at the wound site and enhance angiogenesis by activating the HIF-1α/VEGFA signaling pathway. The hydrogel offers a stable microenvironment that extends the retention time and preserves the biological activity of EVs at the site of injury, thereby synergistically accelerating diabetic wound healing. Compared to conventional diabetic wound care, this approach promotes faster healing and significantly reduces the risks of infection and amputation.	[18]

Note: PLGA-PEG-PLGA, Poly(Lactic-co-Glycolic Acid)-block-Poly(Ethylene Glycol)-block-Poly(Lactic-co-Glycolic Acid); GelMA, Gelatin Methacryloyl; BCG, Bacille Calmette-Guérin.

The integration of EVs with hydrogel systems represents a highly promising strategy for enhancing cancer treatment outcomes. This review explores diverse design strategies, critical determinants, and underlying therapeutic mechanisms of the drug delivery systems consisting of hydrogels. Emphasis is placed on elucidating the synergistic interactions between hydrogel matrices and EVs, rather than evaluating each component in isolation.

## 2 Characteristics and Classification of Hydrogels

### 2.1 Basic Characteristics of Hydrogels

#### 2.1.1 Excellent Biocompatibility

**Figure 1:**
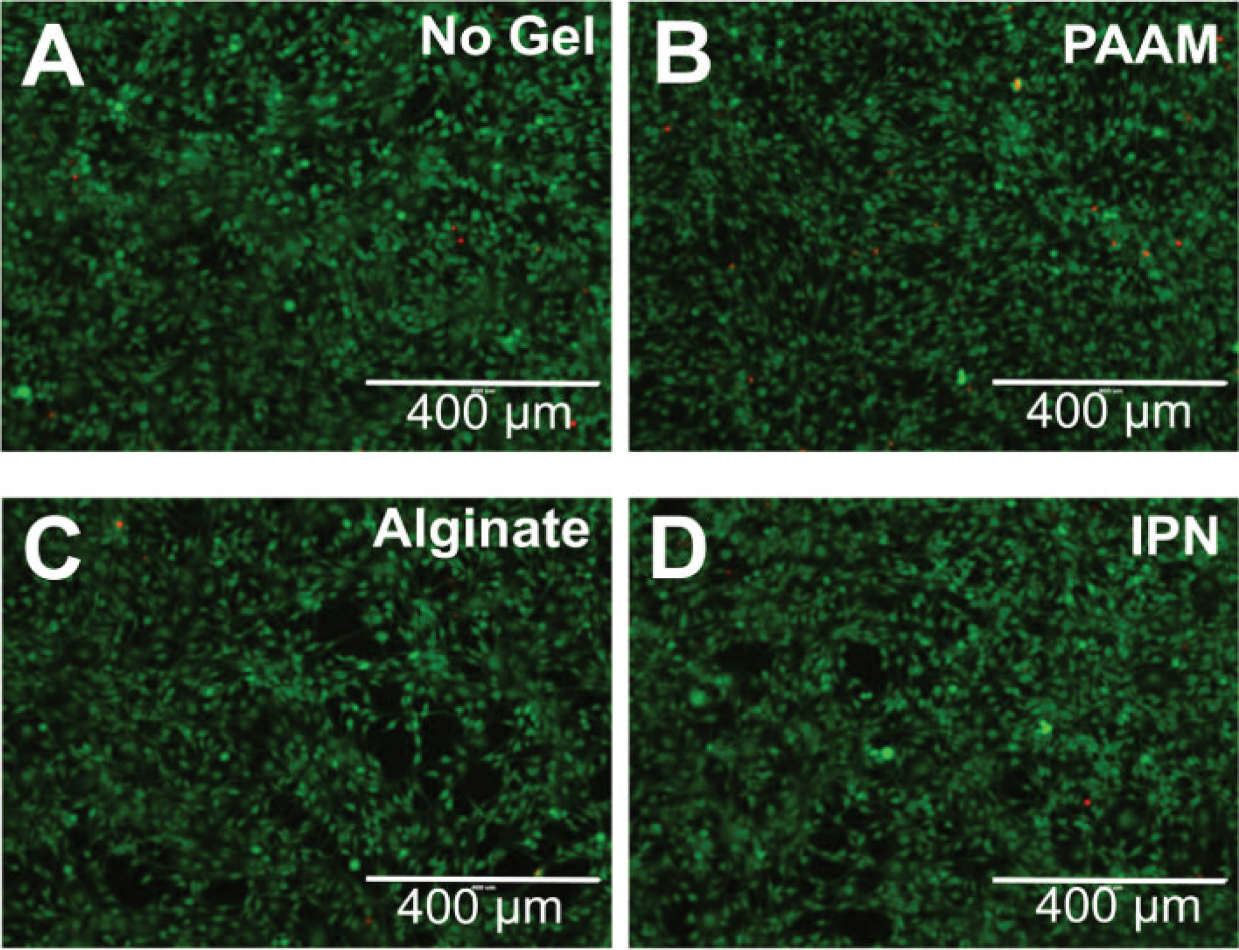
Darnell et al. [19] exposed mesenchymal stem cells to a hydrogel with excellent biocompatibility for 3 days, and these cells maintained a high survival rate. (**A**) Complete DMEM control (no gel); (**B**) Pure PAAM gel; (**C**) Pure Alginate Gel; (**D**) IPN Gel

**Figure 2:**
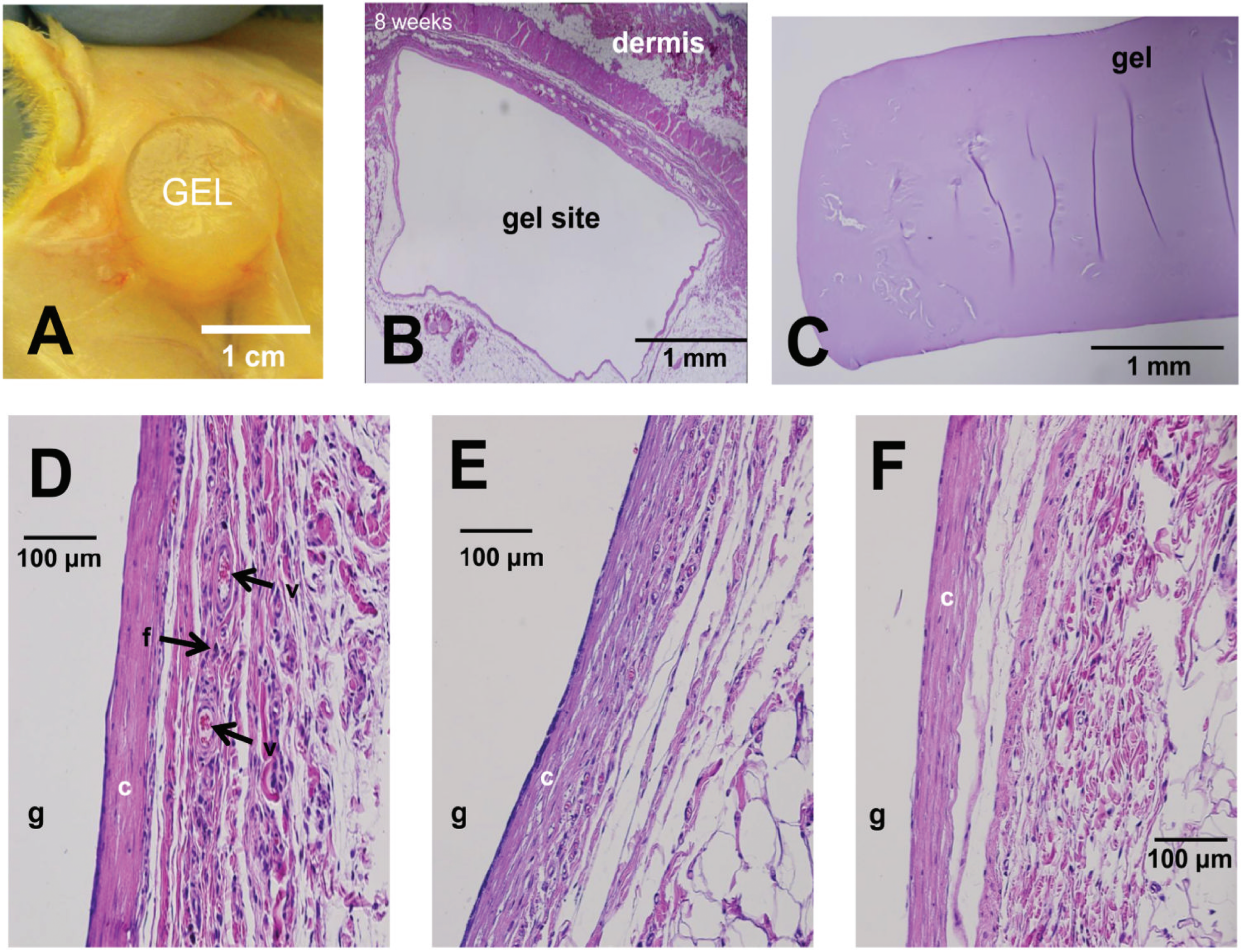
Darnell et al. [19] injected the hydrogel developed by their team subcutaneously into rats. Eight weeks later, no immune cells had infiltrated the surrounding tissues of the hydrogel. (**A**) View of hydrogel in encapsulated subcutaneous pocket, 8 weeks post-implantation. (**B**–**F**) Hematoxylin–Eosin-stained paraffin-embedded sections of the hydrogel implantation site after 8 weeks *in vivo*. Hydrogel location labeled ‘g’, collagen layer labeled ‘c’, active fibroblasts labeled ‘f’, larger blood vessels labeled ‘v’

Biocompatibility is crucial for the biomedical application of hydrogels, as it encompasses the biological, physical, and chemical interactions between these materials and living systems. Hydrogels are recognized for their exceptional biocompatibility in this context. Therefore, many types of hydrogels have been developed by people (Table 2). For example, Darnell et al. developed an alginate/polyacrylamide hydrogel that demonstrated excellent biocompatibility. After exposing mesenchymal stem cells to this hydrogel for 3 days, the cells maintained high viability [19] (Fig. 1). These hydrogels typically contain components such as polysaccharides (e.g., hyaluronic acid, chitosan) and proteins (e.g., collagen, gelatin), which closely mimic natural polymers in biological tissues. Hyaluronic acid, an important element of the extracellular matrix, is present in hydrogels in a structure that closely mimics its natural form in the body. This mimics the extracellular matrix microenvironment and provides an optimal living space for cells. Chitosan’s positive charge interacts with the negative charge of cell surfaces, promoting adhesion and proliferation. Its structure, similar to certain natural polysaccharides, helps it evade immune recognition as a foreign body, thereby reducing the risk of immune rejection [20,21]. Darnell et al. subcutaneously injected the hydrogel developed by their team into rats and performed histological analysis on the surrounding tissues 8 weeks later. The results showed no immune cell infiltration around the hydrogel [19] (Fig. 2). When hydrogels are implanted into the body for tissue repair, surrounding cells naturally adhere to and proliferate on their surface, as if they were growing in their familiar “home”. Importantly, the material does not induce cell apoptosis or dysfunction, which ensures a stable foundation for subsequent therapeutic interventions. For instance, in skin wound repair, hydrogels promote the migration and proliferation of skin cells, accelerate wound healing, and support normal metabolic and functional activities, thereby providing a stable foundation for subsequent therapeutic interventions [11].

**Table 2:** Comparison of different types of hydrogels

Hydrogel Type	Biocompatibility Mechanism	Supporting Studies/Examples	References
Alginate/Polyacry lamide (IPN Gel)	Mimics extracellular matrix (ECM) structure, low immune recognition naturally due to polysaccharide components.	Darnell et al. showed mesenchymal stem cells maintained 90% viability after 3 days of exposure; no immune cell infiltration was observed in rat subcutaneous implants after 8 weeks.	[19]
Chitosan hydrogel	Positive charge cell adhesion; structural similarity to natural polysaccharides reduces immune response.	Chitosan’s interaction with negatively charged cell membranes promotes tissue repair, as seen in studies where it accelerated wound healing without inducing inflammation.	[21]
Extracellular matrix hydrogel	Natural ECM component; interacts with cell surface receptors (e.g., CD44) to promote cell adhesion and proliferation.	Extracellular matrix hydrogels support skin cell migration in wound healing.	[11]

Note: IPN, Interpenetrating Polymer Network.

#### 2.1.2 Controllable Swelling and Drug Release Characteristics

**Figure 3:**
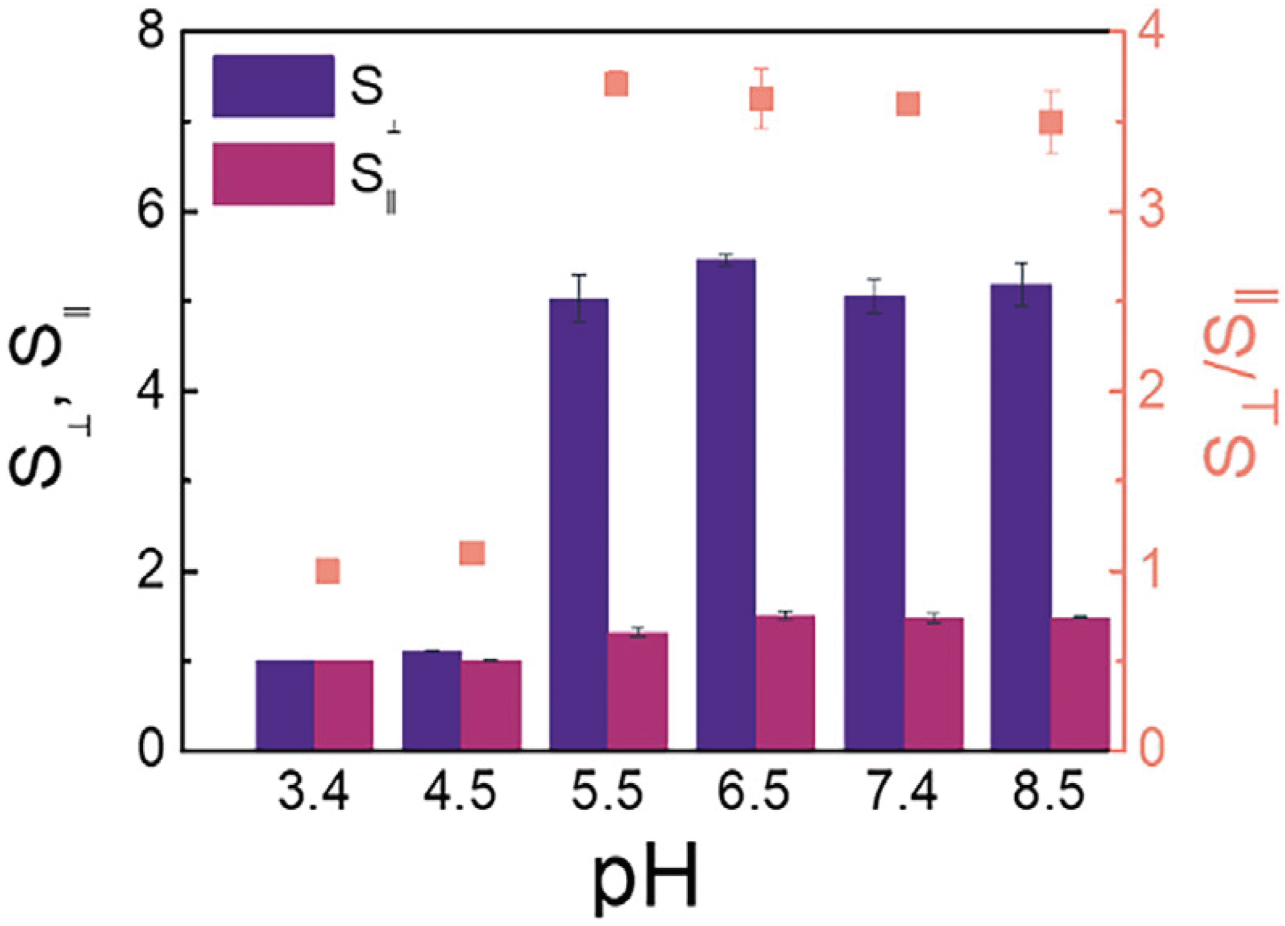
The PLGA composite hydrogel [24] composed of α-helical peptides developed by Zhang et al. exhibits different axial (S_⊥_) and radial (S_Ⅱ_) swelling properties under the influence of different pH values

The swelling properties of hydrogels are dynamic and can be precisely regulated based on changes in external environmental factors [22]. Temperature, pH, and ionic strength are key factors that influence hydrogel swelling, with temperature and pH playing particularly important roles. An example of this is temperature-sensitive hydrogels, such as poly(N-isopropyl acrylamide) (PNIPAm), which integrate both hydrophilic and hydrophobic groups into their molecular structure. When the ambient temperature is below a specific critical solution temperature (LCST), the hydrogen bonds between hydrophilic groups and water molecules predominate, causing water molecules to enter the gel network and resulting in significant swelling. When the temperature exceeds the LCST, hydrophobic interactions within the polymer chain intensify, breaking hydrogen bonds and forcing water molecules out of the network. As a result, the hydrogel shrinks rapidly, its volume decreases sharply, and it transforms into a gel-like state [23]. The Poly(Lactic-co-Glycolic Acid) (PLGA) composite hydrogel, composed of alpha-helical polypeptides and developed by Zhang et al., demonstrated significant swelling in response to pH, showing remarkable potential as an embolic agent [24] (Fig. 3). This temperature-sensitive feature can be leveraged in tumor therapy. Given the higher temperature and acidic microenvironment within tumor tissues, hydrogels can respond to these changes upon reaching the target site, swelling or shrinking accordingly to trigger drug release. This ensures targeted drug delivery, minimizing damage to surrounding healthy tissues [25].

**Figure 4:**
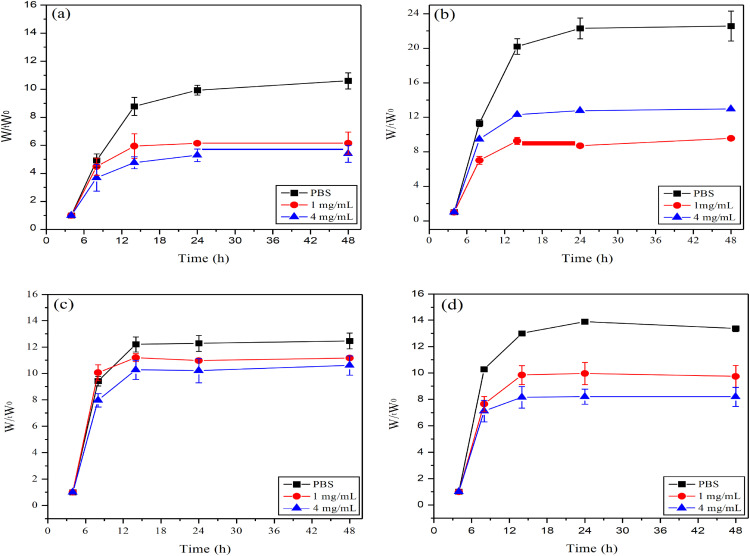
In Elshaarani et al. [26], four different ratios of hydrogels were respectively added to PBS solution with a pH value of 7.9, glucose solution at 1 mg/mL, and glucose solution at 4 mg/mL. The swelling ratios of these four hydrogels were obtained. (**a**): CS(Chitosan)0%PBA(Phenylboronic acid)10%; (**b**): CS2.5%PBA10%; (**c**): CS5%PBA10%; (**d**): CS10%PBA10%. W_t_: refers to the weight of the hydrogel at a specific time point (such as a certain moment after being treated with different glucose concentrations). W_0_: refers to the initial weight of the hydrogel (usually the weight in a dry state)

The drug release behavior of hydrogels is closely linked to their swelling properties, with both factors working synergistically to enhance therapeutic efficacy. In a study by Elshaarani et al., a polyacrylamide-co-3-acrylamido-phenylboronic acid-chitosan grafted maleic acid (P(AM-co-AAPBA-co-CSMA)s) hydrogel was synthesized using polyethylene glycol diacrylate (PEGDA) as the cross-linking agent. The hydrogel’s swelling behavior can be modulated in response to varying glucose concentrations, enabling glucose sensing and controlled drug release due to differences in binding affinity [26] (Fig. 4). For instance, in the swelling phase, at lower temperatures, drug molecules are evenly dispersed within the hydrogel network, akin to goods placed on a warehouse shelf. As the temperature rises and the hydrogel shrinks, the gel network becomes more compact, creating internal pressure that accelerates the outward diffusion of the drug, similar to goods being released from a warehouse once the door opens [27]. In addition to temperature sensitivity, the chemical structure of hydrogels can be engineered to incorporate specific bonds or groups that are responsive to tumor-expressed enzymes. This allows for enzyme-triggered drug release, enhancing the precision and efficiency of drug delivery and enabling personalized treatment strategies for tumors [28].

### 2.2 Classification of Hydrogels

#### 2.2.1 Natural Polymer Hydrogels

Natural polymer hydrogels, particularly hyaluronic acid-based hydrogels, are crucial in biomedicine because they derive from biological sources [29]. Hyaluronic acid, a naturally occurring polysaccharide that is abundant in the human body, exhibits exceptional biocompatibility. It can specifically interact with cell surface receptors, fostering a near-natural microenvironment for cells, thus minimizing the risk of immune rejection. Its distinctive linear, branchless polymer structure provides the hydrogel with exceptional water retention, akin to creating a “reservoir” within tissue, ensuring optimal hydration and preserving normal cell morphology and function [29]. Recent advancements in tumor therapy have seen research teams combine hyaluronic acid hydrogels with the chemotherapy drug doxorubicin to develop a novel drug delivery system [30]. The precise control of cross-linking degree allows this hydrogel to achieve gradual and sustained drug release, maintaining effective drug concentrations at the tumor site over an extended period. It demonstrated remarkable antitumor effects on tumor growth in animal models, with minimal damage to the normal tissues surrounding the tumors. These results suggest that hyaluronic acid-based hydrogels have significant potential for targeted tumor therapies, potentially altering clinical strategies in cancer treatment.

Natural polymer-derived hydrogels, aside from those based on hyaluronic acid, present unique functional benefits. Collagen, a key structural component of the extracellular matrix (ECM), plays a crucial role in preserving tissue architecture and biomechanical integrity. Hydrogels formulated from collagen naturally facilitate cellular attachment and movement, owing to integrin-recognized motifs such as the arginine–glycine–aspartic acid (RGD) sequence. These characteristics render collagen-based hydrogels particularly suitable for regenerative medicine and tissue engineering applications, such as dermal wound healing and cartilage regeneration [31]. Alginate, a naturally occurring polysaccharide derived from brown seaweed, undergoes rapid gel formation through ionic cross-linking, commonly initiated by divalent calcium ions (Ca^2+^). This gentle gelation process allows for the encapsulation of viable cells and labile bioactive agents, supporting its widespread use in wound healing and cell delivery applications [32]. Gelatin, a hydrolyzed derivative of collagen, preserves the compatibility of its source with biological systems while adding temperature-sensitive behavior. Its sol–gel transition near physiological temperature makes it well-suited for injectable therapies. As a result, gelatin is widely utilized in localized drug delivery and as a scaffold or filler for tissue regeneration [33]. The diversity in origin, molecular structure, and biological functionality of these polymers underscores the broad therapeutic potential of natural hydrogel platforms.

#### 2.2.2 Synthesis of Polymeric Hydrogels

Synthetic polymer hydrogels, with customized chemical structures and properties, offer significant potential across various biomedical applications. One notable example is Poly(Lactic-co-Glycolic Acid) (PLGA) hydrogels [34], typically synthesized through the ring-opening polymerization of lactic acid and glycolic acid. The degradation rate of these hydrogels, ranging from weeks to months, can be precisely controlled by adjusting the ratio of lactic acid and glycolic acid to meet specific treatment requirements [35]. This degree of control enables the precise and gradual release of anti-tumor drugs, ensuring therapeutic stability and prolonging efficacy. Tsai et al. developed an Adriamycin-loaded PLGA-alginate hydrogel for intratumoral injection. During degradation, the hydrogel released a high concentration of the drug, demonstrating effective thermochemotherapy both *in vitro* and *in vivo* [36]. Cancer cell proliferation was effectively inhibited, with minimal systemic adverse reactions. This offers new hope for patients with advanced tumors and underscores the promising potential of synthetic polymer hydrogels in targeted tumor therapy.

#### 2.2.3 Intelligent Responsive Hydrogel

The smart responsive hydrogel can sense and respond simultaneously, detecting subtle changes in the tumor microenvironment and responding accordingly. pH-responsive hydrogels are particularly effective. Tumor tissue typically has an acidic microenvironment due to increased metabolic activity, with a pH that is 0.5–1.5 units lower than that of normal tissue pH-responsive hydrogels can specifically target this acidic environment [37]. pH-sensitive groups, such as carboxyl and amino groups, are incorporated into the molecular structure of these hydrogels. In neutral conditions, the hydrogel maintains a stable and compact structure due to stable intramolecular and intermolecular hydrogen bonding. When exposed to the acidic tumor microenvironment, these sensitive groups undergo protonation or deprotonation, disrupting hydrogen bonds. The hydrogel network structure rapidly “unlocks,” facilitating controlled release of encapsulated anti-tumor drugs and promoting targeted delivery to the tumor site. A pH-responsive hydrogel composed of chitin nanofibers and alginate dialdehyde (CHADA) was designed for adriamycin delivery. The doxorubicin (DOX)@CHADA hydrogel showed a faster and more pronounced release of DOX at pH 5.5 and 6.5, while release was significantly reduced at pH 7.4. Under the mildly acidic conditions of pH 5.5, chemical bonds in the hydrogel, such as Schiff bases and ionic bonds, were partially cleaved, facilitating DOX release. After 14 days of treatment with the DOX@CHADA hydrogel, tumor regression and necrosis were markedly more pronounced compared to the free DOX group. The hydrogel significantly enhanced the efficacy of antitumor drugs, improving their utilization and killing effect on tumor cells. This approach offers a promising strategy to address tumor drug resistance [38].

In addition to pH-responsive systems, various types of stimuli-responsive hydrogels have been developed. Among them, temperature-responsive hydrogels are among the most widely studied. Poly(N-isopropylacrylamide) (PNIPAAm) hydrogels remain in a swollen state at lower temperatures due to hydrogen bonding between water molecules and polymer chains. When the temperature exceeds its lower critical solution temperature (LCST), hydrogen bonds are disrupted, hydrophobic interactions dominate, and the hydrogel undergoes volume contraction [23]. Enzyme-responsive hydrogels leverage enzymatic catalysis of specific substrates to initiate structural or functional changes. For instance, hydrogels that utilize peptide-based crosslinking agents can be engineered to respond to specific enzymatic activity. When exposed to target enzymes, these peptide linkers are enzymatically cleaved, compromising the integrity of the hydrogel network. This structural disruption leads to swelling or disintegration, which can be harnessed for enzyme sensing or for initiating drug release in a controlled manner. In the context of triple-negative breast cancer (TNBC), responsive delivery systems are tailored to interact with the tumor microenvironment. Elevated levels of matrix metalloproteinases, particularly MMP-2 and MMP-9, commonly observed in TNBC, catalyze the breakdown of the hydrogel scaffold, thereby facilitating the regulated release of therapeutic agents stored within the matrix. This localized delivery minimizes systemic toxicity and enhances therapeutic efficacy [28].

### 2.3 Limitations of Hydrogels for Drug Delivery Alone

Despite the many advantages of hydrogels in drug delivery, they have notable limitations when used alone as carriers. Hydrogels primarily rely on passive diffusion for drug release, which is slow and lacks precise control. In the complex and dynamic *in vivo* environment, hydrogels often fail to meet the specific requirements of tumor tissues [39]. When delivering hydrophilic drugs, the interaction between the hydrogel’s hydrophilic groups and water molecules increases diffusion resistance, slowing drug release. Consequently, the drug release rate may not meet therapeutic needs, reducing the achievement of effective drug concentrations at the tumor site and impairing therapeutic efficacy [40]. Hydrophobic drugs have poor solubility in hydrogels and tend to aggregate, hindering uniform dispersion and effective release, thus limiting their antitumor efficacy [28].

Furthermore, achieving the correct balance between hydrogel stability *in vivo* and degradation rate is challenging. If the stability is too high, hydrogels may persist in the body after completing the drug delivery, potentially causing inflammation or other adverse effects. Conversely, if the degradation rate is too rapid, premature drug release may occur, compromising drug targeting, wasting the drug, and potentially harming healthy tissues. Furthermore, hydrogels on their own lack active targeting capabilities and cannot selectively target tumor tissues; drugs may therefore affect not only tumor tissues but also healthy organs and tissues, increasing the burden on patients and causing chemotherapy-related side effects such as nausea, vomiting, and hair overcome these limitations, it is essential to integrate additional functional components with hydrogels [41]. The emergence of extracellular vesicles offers a solution, and their combination is expected to open a new chapter in precision cancer therapy.

## 3 Overview of Extracellular Vesicles

EVs are nanoscale, lipid-bilayer-enclosed structures released by cells and found throughout the body, where they play essential roles in both normal physiology and disease development. These particles are highly diverse and are typically divided into three main categories: exosomes, microvesicles, and apoptotic bodies (Table 3). Exosomes, which range in size from approximately 50 to 200 nm, arise from the endosomal system through the inward budding of cellular membranes. In contrast, microvesicles are larger—measuring between 100 and 1000 nm—and are formed by direct outward protrusion of the plasma membrane. Apoptotic bodies, the largest subtype (1–5 μm), are produced during the late stages of programmed cell death (apoptosis) [42]. The formation of EVs involves various molecular mechanisms and intracellular signaling pathways. For example, exosome generation is linked to ESCRT complexes, tetramembrane proteins, and sphingomyelinase [43].

**Table 3:** Comparison of different types of extracellular vesicles (EVs)

EV type	Size range	Origin mechanism	Biological functions in tumor therapy	References
Exosomes	30–150 nm	Derived from endosomal system; fusion of multivesicular bodies (MVBs) with cell membrane.	Transport tumor-associated antigens to activate immune cells (e.g., dendritic cells). Carry miRNAs and mRNA to regulate tumor cell drug proliferation and resistance. Natural targeting to tumor microenvironment via surface proteins.	[50]
Microve sicles	100–1000 nm	Direct budding from the cell membrane, regulated by cytoskeletal proteins (e.g., actin) and annexins.	Promote tumor angiogenesis by delivering pro-angiogenic factors (e.g., VEGF). Suppress anti-tumor immune responses by carrying immunosuppressive molecules (e.g., TGF-β, PD-L1). Facilitate tumor cell migration and metastasis via matrix metalloproteinases (MMPs).	[54]
Apoptotic bodies	1–5 μm	Formed during programmed cell death (apoptosis). released when the cell membrane breaks down.	Induce immune tolerance by delivering apoptotic antigens to phagocytes. Potentially carry anti-tumor drugs when engineered as delivery vehicles. Regulate tumor microenvironment via the release of pro-inflammatory or anti-inflammatory factors.	[42]

**Figure 5:**
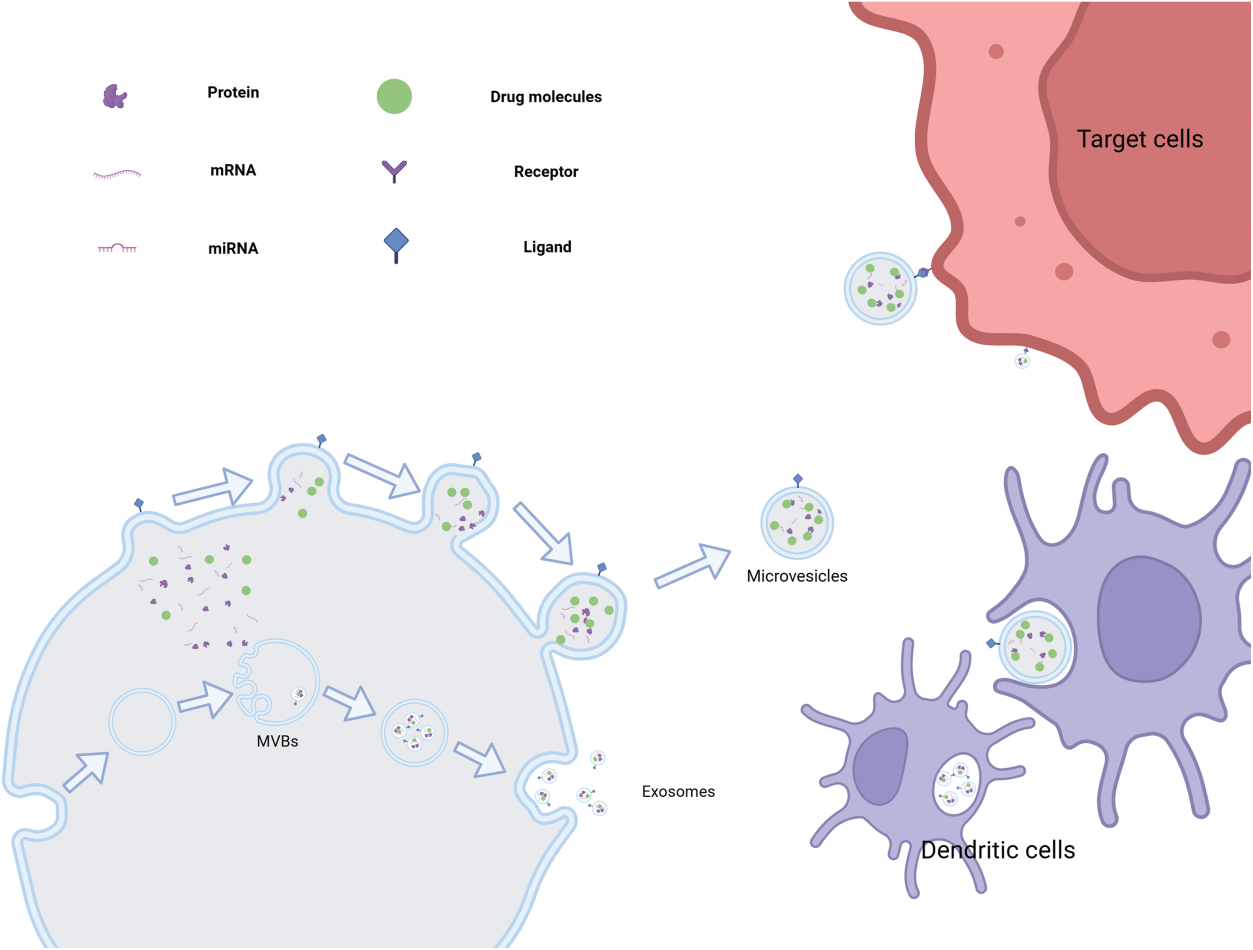
The various generation methods of extracellular vesicles and their potential for targeted drug delivery. (This picture was drawn by biorender. Agreement number: ML28Q5HFJR)

EVs are enriched with biomolecules, including proteins, nucleic acids (such as mRNA, miRNA, and DNA), and molecules are delivered to target cells, where they facilitate intercellular communication and contribute to both physiological and pathological processes. In the context of disease, EVs are closely associated with tumor initiation and progression, promoting tumor cell proliferation, migration, angiogenesis, and immune molecular markers carried by EVs hold potential as a basis for tumor diagnosis [44]. In neurodegenerative diseases, EVs can transfer pathogenic proteins, such as beta-amyloid in Alzheimer’s disease and alpha-synuclein in Parkinson’s disease [45]. EVs hold significant promise in therapeutic applications. As drug delivery vehicles, EVs exhibit excellent biocompatibility, effectively protecting drugs from degradation while enabling precise, targeted delivery. This capability can significantly enhance drug efficacy and potentially create new strategies for disease treatment [46] (Fig. 5).

### 3.1 The Origin and Type of Extracellular Vesicles

#### 3.1.1 Exosomes

Endosomal vesicles containing proteins, nucleic acids (e.g., mRNA and miRNA), lipids, and other bioactive molecules are initially generated in cells. These vesicles are then enclosed within multivesicular bodies (MVBs), whose transport and maturation are regulated by various proteins. These complexes, the endosomal sorting complex required for transport (ESCRT) protein family acts as ‘porters’ sorting and packaging biomolecules while directing the movement of MVBs to the cell membrane [47]. During this process, the ESCRT complex, through the coordinated action of its subunits, recognizes and binds specific biomolecules, sorting them into polyvesicles to ensure that each polyvesicle carries molecular cargo with a defined composition and function [48]. Upon reaching the membrane, the outer membrane of the MVB fuses with the cell membrane, releasing the internal vesicles into the extracellular released vesicles are called exosomes.

Exosomes typically range from 30 to 150 nm in diameter, a size that enables them to navigate the complex biological environment flexibly. Their surfaces are rich in membrane proteins, which confer unique biological functions and enable target recognition. Exosomes secreted by tumor cells are particularly enriched in specific membrane proteins, allowing them to recognize signal molecules in the tumor microenvironment. This facilitates the targeted movement of exosomes toward neighboring or distant tumor cells, enabling long-distance cell communication [49]. Exosomes carry nucleic acids, particularly miRNA, which can enter recipient cells and modulate gene expression, influencing key biological processes such as cell proliferation, differentiation, and apoptosis. As a result, exosomes play a crucial role in tumor initiation, progression, metastasis, and drug resistance, making them important targets and tools for therapeutic intervention in cancer research [50].

Exosomes can also transport other bioactive molecules, such as growth factors, cytokines, and enzymes, which play critical roles in cell-to-cell communication and the regulation of the tumor, they highlight their significant role in tumor biology [51].

#### 3.1.2 Microvesicles

Unlike exosomes, microvesicles are generated through a more direct process. These extracellular vesicles form by budding off directly from the cell membrane. During this process, cytoskeletal proteins help maintain local deformation of the membrane and provide structural support for the budding event. Additionally, annexin and other associated proteins play a crucial regulatory role by promoting the gradual bulging of the cell membrane in specific regions, ultimately resulting in the detachment of vesicles from the parent cell [52].

Microvesicles have a larger size range, typically from 100 to 1000 nm in diameter, and are often referred to as ‘giants’ relative to exosomes. Microvesicles not only contain components of the cell membrane but also incorporate cytoplasmic materials, including a variety of proteins, lipids, and small amounts of bioactive molecules such as nucleic acids. These contents give microvesicles significant potential for intercellular communication and functional regulation [53]. In the tumor microenvironment, microvesicles released by tumor cells act as “mobile signaling stations”. They transmit specific biological information from tumor cells to surrounding stromal and immune cells, inducing phenotypic changes in stromal cells, promoting angiogenesis, and supplying nutrients essential for tumor growth [54]. Conversely, microvesicles can modulate immune cell functions by suppressing anti-tumor immune responses, including inhibiting T cell proliferation and activation and reducing NK cell cytotoxicity. This enables tumor cells to evade immune surveillance and facilitates tumor progression [55].

### 3.2 The Role of Extracellular Vesicles in Tumor Therapy

#### 3.2.1 Immunoregulatory Function

Extracellular vesicles, particularly exosomes and microvesicles secreted by tumor cells, play a crucial role in tumor immune membranes display numerous tumor-associated in the immune system, they are efficiently internalized by antigen-presenting cells, including dendritic cells [56]. The tumor antigens captured by dendritic cells are processed, expressed on their surface, and presented for the activation of cytotoxic T lymphocytes and natural killer leads to an efficient antitumor immune response.

Zhang et al. successfully isolated exosomes secreted by melanoma cells and engineered autologous cell-derived exosomes with MnExo@cGAMP [57]. The study included *in vitro* experiments demonstrating that exosomes derived from melanoma cells significantly promoted dendritic cell (DC) maturation, evidenced by increased expression of surface markers CD80 and CD86. Furthermore, co-culture of T cells with these mature DCs resulted in a marked expansion of the CD8^+^ T lymphocyte population. This was accompanied by elevated secretion of pro-inflammatory cytokines, including TNF-α and IL-6, indicating that MnExo@cGAMP possesses considerable therapeutic potential in melanoma immunotherapy. The significant inhibition of tumor progression underscores the potential of extracellular vesicles to elicit anti-tumor immune responses, reinforcing their promise as a novel cancer immunotherapy strategy.

However, tumor cells also possess immune-evasive properties. Tumor-derived vesicles can carry immunosuppressive molecules, such as programmed death ligand 1 (PD-L1) and transforming growth factor-β (TGF-β), which inhibit immune cell activity and facilitate immune evasion by tumor cells [58,59]. Future research may focus on modifying these vesicles, such as using gene-editing technologies to knock out immunosuppressive molecules on their surfaces or employing targeted antibodies to block their effects. These strategies could reverse immune suppression, enabling the immune system to more effectively target and eliminate tumor cells.

#### 3.2.2 Potential of Piggy-Back Drugs

Extracellular vesicles have a unique bilayer membrane structure, creating an independent and stable internal space that provides an ideal environment for carrying various anti-tumor drugs [60]. Both small-molecule chemotherapy drugs, such as doxorubicin and paclitaxel, and large-molecule biologics, including nucleic acid drugs (e.g., siRNA, mRNA) and protein therapeutics (e.g., antibodies, cytokines), can be efficiently encapsulated within these vesicles through various loading strategies [61,62]. Small-molecule drugs take advantage of the concentration gradient across the vesicle membrane, entering the vesicle via passive diffusion. The acidic tumor microenvironment can alter the surface charge and membrane stability of extracellular vesicles, causing structural changes that enhance their ability to penetrate the extracellular matrix and interact with tumor cells. Additionally, components of the extracellular matrix, such as collagen and fibronectin, may bind to specific proteins on the vesicle surface, functioning as “guides” that direct the vesicles along protein fibers toward tumor cells.

It is important to note that the drug loading capacity of extracellular vesicles is not unlimited and depends on certain characteristics of the drug, including size, charge, hydrophilicity, and hydrophobicity. In general, drug molecules or nanoparticles with diameters between 10 and 100 nm are most efficiently loaded into vesicles, such as exosomes. A drug with an appropriate charge can interact with the charged groups on the vesicle membrane, facilitating the loading process. For instance, a positively charged drug can attract negatively charged phospholipids on the vesicle membrane, enhancing drug entry [63]. Additionally, the hydrophobicity of the drug influences its stability and release properties within the vesicle [64]. It is believed that by continuously optimizing loading methods and conditions, the drug-loading capacity of extracellular vesicles can be maximized, enabling efficient drug delivery and enhancing the efficacy of tumor therapy.

#### 3.2.3 Potential for Targeted Drug Delivery

The surface of extracellular vesicles is enriched with a variety of specific membrane proteins and lipid components, enabling precise recognition and targeted binding to tumor tissues based on unique molecular markers present on tumor cells. For instance, the epidermal growth factor receptor (EGFR) is highly expressed on the surface of many epithelial-derived tumor cells, and certain stem cell-derived vesicles carry ligands that specifically bind to EGFR. This interaction resembles the precise “key-and-lock” mechanism. This interaction allows extracellular vesicles to target and bind specifically to tumor cells, thereby efficiently delivering loaded drugs into the interior of the cells for targeted therapy and minimizing damage to normal tissues [65]. When the extracellular vesicle approaches the tumor cell, a specific membrane protein on its surface interacts with the corresponding receptor on the tumor cell, triggering both vesicle-cell binding and a cascade of intracellular signaling pathways that enhance the uptake of vesicle contents by tumor cells [66].

Furthermore, as research on the tumor microenvironment progresses, it has become evident that factors such as extracellular matrix composition, pH, and osmotic pressure around tumor tissues significantly differ from those in normal tissues. Extracellular vesicles are capable of sensing these subtle changes and traversing the complex extracellular matrix barrier with the assistance of chemokine gradients and biophysical signals. This ability to detect microenvironmental alterations enhances the targeting of drug delivery to tumor cells, offering new strategies for overcoming tumor drug resistance and improving treatment outcomes in advanced cancer patients [67]. The acidic tumor microenvironment can alter the surface charge and membrane stability of extracellular vesicles, causing structural changes that enhance their ability to penetrate the extracellular matrix and interact with tumor cells. Furthermore, components of the extracellular matrix, such as collagen and fibronectin, may bind to specific proteins on the vesicle surface, acting as ‘guides’ that direct the vesicles along protein fibers toward tumor cells [68].

### 3.3 Limitations of Extracellular Vesicles Application

Although extracellular vesicles have demonstrated significant advantages in cancer therapy, their clinical application still faces several bottlenecks that need to be addressed. On one hand, the preparation process for extracellular vesicles remains complex and costly, which severely limits their large-scale production and widespread use. For instance, the methods for isolating exosomes include ultracentrifugation, density gradient centrifugation, polyethylene glycol precipitation, ultrafiltration, immunomagnetic beads, and exclusion chromatography. Each of these techniques has limitations. While ultracentrifugation is the most commonly used method, it is labor-intensive, time-consuming, and requires specialized equipment, often resulting in exosomes with unsatisfactory purity [69]. Although the immunomagnetic bead method can preserve exosome integrity and ensure high specificity, non-neutral pH and non-physiological salt concentrations can negatively affect exosome biological activity, hindering subsequent experiments [70].

On the other hand, the stability of extracellular vesicles *in vivo* is a critical concern. As natural nanoscale structures, they are highly susceptible to degradation by various enzymes, proteins, and the complex biological environment during circulation. This degradation, along with clearance by the immune system, can lead to premature drug leakage, preventing accurate delivery to the tumor site and significantly reducing therapeutic efficacy [67]. Enzymes in the tumor microenvironment, such as matrix metalloproteinases, can degrade the vesicle membrane, compromising its integrity and resulting in drug loss. Additionally, antigens or molecular patterns on the vesicle surface can be recognized as foreign by macrophages via phagocytosis, preventing the drugs from effectively reaching tumor cells. Interdisciplinary approaches that design carriers with high hydrophilicity, excellent biocompatibility, stability, and controlled release capabilities could fully harness the potential of extracellular vesicles. Undoubtedly, hydrogels are an ideal candidate for carriers.

## 4 Construction of Drug Delivery Systems Consisting of Hydrogels Loaded with Extracellular Vesicles

### 4.1 Construction Strategy and Methods

Recent advances in tumor treatment have focused attention on drug delivery systems utilizing EVs. These vesicles inherently possess targeting abilities, enabling precise identification of tumor cells and enhancing the accuracy of targeting and drug action [71]. Additionally, EVs can carry a variety of therapeutic agents, offering versatile functions high flexibility. Hydrogels, with favorable physical and chemical properties, serve as excellent carriers for EVs. Their biocompatibility ensures *in vivo* safety, while their porous swith tructure facilitates efficient drug loading, and their adjustable properties enable sustained, long-term drug release. The integration of these two technologies overcomes the limitations of each individual method in drug delivery [72]. However, an ongoing challenge lies in effectively loading EVs into hydrogels. This review examines two primary methods—physical blending and chemical cross-linking—and evaluates their mechanisms for successfully encapsulating EVs into hydrogels for cancer therapeutics.

#### 4.1.1 Physical Blending Method

**Figure 6:**
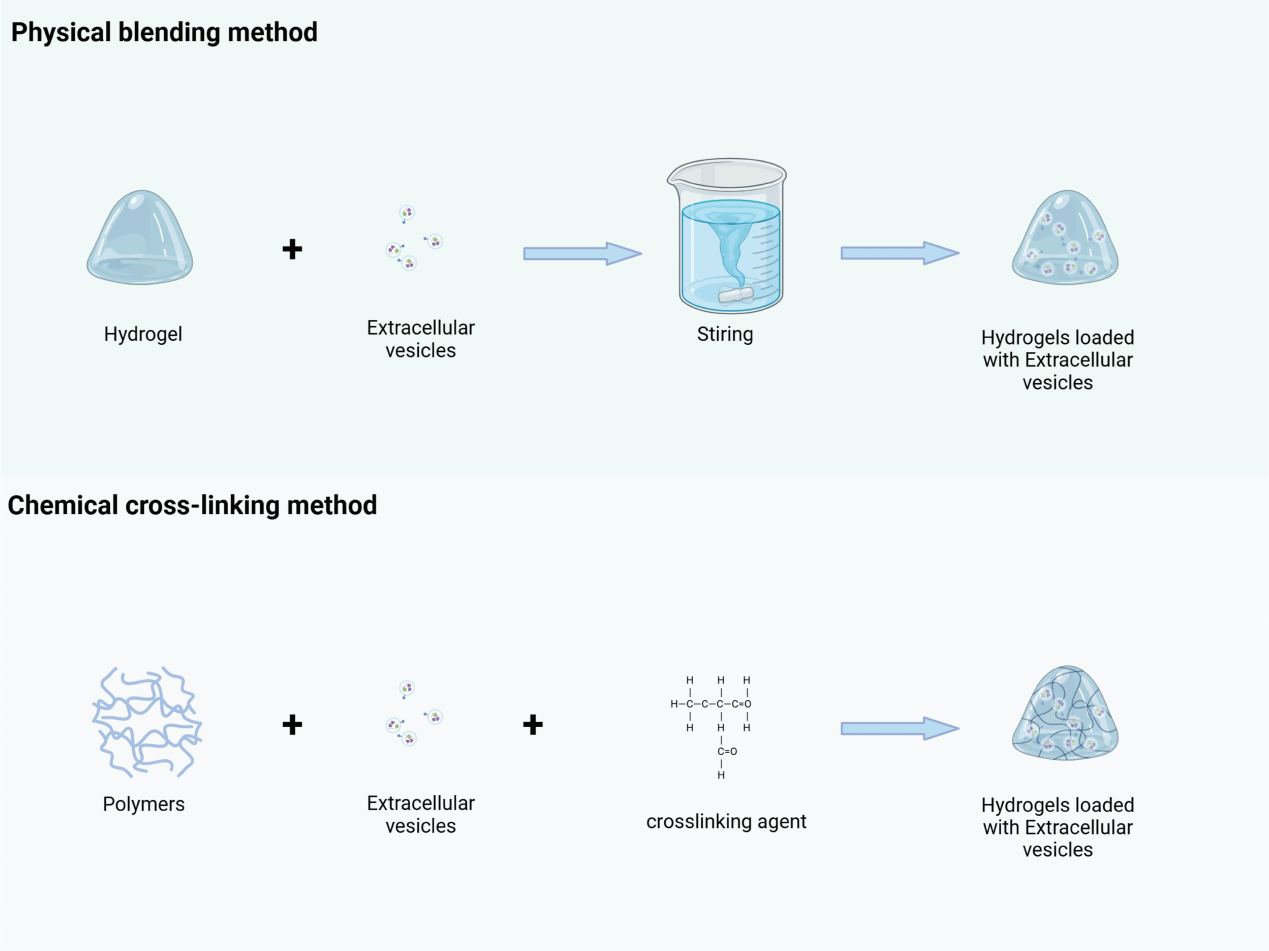
EVs were loaded into the hydrogel through physical blending and chemical crosslinking methods. Physical blending method: Pre-prepared hydrogels are mixed with EVs under mild conditions (such as low-speed stirring), and EVs are uniformly dispersed and embedded in the three-dimensional network of the hydrogel through weak interactions such as hydrogen bonds and van der Waals forces, forming EV-hydrogel complexes. Chemical crosslinking method: Through the mediation of crosslinking agents (such as polyethylene glycol diacrylate, PEGDA), the hydrogel polymer chains undergo chemical reactions with the active functional groups (such as carboxyl and amino groups) on the surface of EVs, forming covalent bonds to construct structurally stable EV-hydrogel complexes. (This picture was drawn by Biorender. Agreement number: BG28Q5IMHF)

One of the simplest and most direct methods for constructing a drug delivery system consisting of hydrogels loaded with extracellular vesicles is the physical blending method (Fig. 6). This approach involves mixing pre-prepared hydrogel with extracellular vesicles under specific mild conditions using mechanical stirring, ultrasonic treatment, or other techniques. Taking polyethylene glycol (PEG) hydrogel and macrophage-derived exosomes as an example, firstly, PEG hydrogel with an appropriate crosslinking degree was prepared by conventional polymerization reaction, and the hydrogel had good hydrophilic and biocompatibility. Exosomes are then isolated from the macrophage culture supernatant using ultracentrifugation, ensuring the preservation of their structural integrity and biological activity. A certain amount of exosomes is then gently dispersed into the prepolymerization solution of PEG hydrogel, and under low-speed stirring, the exosomes are uniformly distributed within the hydrogel system. As the crosslinking reaction progresses, the exosomes become firmly embedded within the hydrogel, forming the drug delivery system [73]. During the stirring process, both the stirring speed and duration should be carefully controlled. Excessively high stirring speeds can lead to the rupture of exosomes, while insufficient stirring time may result in uneven dispersion, compromising the performance of the final drug delivery system. Additionally, this method relies on weak intermolecular interactions, including hydrogen bonding and van der Waals forces. As a result, the binding stability between EVs and hydrogels is relatively low. *In vivo*, EVs are susceptible to premature detachment due to enzymatic degradation or osmotic pressure fluctuations, resulting in uncontrolled drug release. Furthermore, drug loading is constrained by limited physical embedding space, making high-capacity loading challenging [74].

This physical blending method depends on weak intermolecular interactions, such as hydrogen bonding and van der Waals forces, to promote the initial binding of the hydrogel to the extracellular vesicles. From an operational standpoint, this method is straightforward, requiring no complex chemical reactions, and utilizes relatively simple experimental equipment and techniques. It can be completed in a short time, significantly improving preparation efficiency. By precisely controlling the mixing ratio, the drug loading can be effectively regulated, providing a foundation for subsequent drug release [74].

#### 4.1.2 Chemical Cross-Linking Method

The chemical cross-linking method utilizes chemical reactions to form stable covalent bonds between the hydrogel and extracellular vesicles, resulting in a highly integrated drug delivery system (Fig. 6). Common cross-linking strategies include agents such as glutaraldehyde and carbodiimide, or direct cross-linking reactions targeting active functional groups on the surface of extracellular vesicles or the hydrogel’s molecular chains. For instance, Qin et al. used poly(ethylene glycol) diacrylate as a cross-linking agent and a composite matrix of thiolized hyaluronic acid, heparin, and gelatin to encapsulate BMSC-derived exosomes. This method activated a reaction between the carboxyl group on the exosome surface and the amino group on the hydrogel, promoting an amidation reaction to form a stable chemical bond [75]. Qin X’s team developed an innovative extracellular vesicle-hydrogel composite system for repairing endometrial injury and restoring fertility. This system involves combining extracellular vesicles from specific cell types with appropriate polymers, which are then delivered to the target site using a double-chamber syringe, along with cross-linking agents. During injection, the active groups on the surface of the extracellular vesicles (e.g., carboxyl and amino groups) chemically interact with corresponding functional groups on the polymer or cross-linking agent, allowing the polymer chains to bind and encapsulate the extracellular vesicles. This process rapidly forms a stable hydrogel structure at the target site, ensuring effective loading [76]. However, crosslinking agents used in this approach, such as glutaraldehyde, may react non-specifically with EV surface proteins, compromising their biological activity and targeting capability. Precise control of reaction parameters—such as temperature and pH—is essential; otherwise, heterogeneous crosslinking may occur. Over-crosslinking can densify the hydrogel network and impede EV release, whereas under-crosslinking may result in premature degradation of the delivery system. Moreover, the complexity of the crosslinking process hampers scalability, and residual crosslinking agents may induce cytotoxic or systemic toxic effects *in vivo*.

Compared to the physical blending method, the drug delivery system created by chemical cross-linking offers greater stability, effectively resisting structural damage in the complex biological environment *in vivo* and minimizing the risk of premature drug leakage. In drug release regulation, the presence of chemical bonds allows for more precise control over the release rate. The degree of cross-linking, as well as the type and concentration of cross-linking agents, can be precisely adjusted to control the kinetic profile of drug release. A study from Indonesia investigated the effect of cross-linking between glutaraldehyde and gelatin on the pore size of hydrogels. As the amount of glutaraldehyde increased, the degree of cross-linking also increased, leading to a reduction in pore size and a significant decrease in the rate of drug release [77].

### 4.2 Key Factors Affecting the Construction

#### 4.2.1 Ratio of Hydrogel to Extracellular Vesicles

The ratio of hydrogel to extracellular vesicles is a critical factor in constructing an effective drug delivery system, as it significantly influences drug encapsulation efficiency, release kinetics, and therapeutic outcomes. Variations in this ratio can lead to marked differences in the physicochemical properties and biological behaviors of the system. The release of membrane vesicles is primarily driven by hydrogel swelling and the diffusion of vesicles within its the hydrogel content predominates in the system, the release dynamics are example, Han et al. developed an injectable PLGA hydrogel containing exosomes derived from dental pulp stem cells and vascular endothelial growth factor (VEGF). With 20% PLGA-PEG-PLGA and a 5 mg/mL exosome concentration, the hydrogel’s dense 3D network provided ample physical sites for drug embedding. The abundance of hydrogel acted as a “storage lattice,” tightly retaining the drug. During the initial stages of drug release, however, the DPSCs-Exo system exhibited significant resistance to drug diffusion, resulting in a slow release 9.9% of the DPSCs-Exo was released within the first 7 days, with continuous release observed over the next 3 weeks, peaking on day 15 [15].

On the contrary, when the ratio of hydrogel to extracellular vesicles is reduced and the relative content of extracellular vesicles is increased, the peak of the drug release curve will shift significantly. As the content of vesicles increases, the hydrogel network must accommodate more vesicles during swelling, which may restrict the swelling process as vesicles occupy space that water molecules would enter, slowing the swelling rate and indirectly affecting vesicle release. Besides, the increased concentration of vesicles enhances their diffusion within the hydrogel network. For example, Xu et al. prepared a gel with a higher exosome concentration, where exosomes were rapidly released within 12 h, followed by a sustained release for the next 60 h [78]. Therefore, the precise regulation of the hydrogel-to-vesicle ratio is essential for achieving efficient and stable drug delivery. In applying this system to tumor treatment, it is crucial to carefully consider the distinct requirements at each treatment stage and precisely adjust the corresponding proportions accordingly.

The drug release profile directly influences the biodistribution of therapeutic agents. Sustained release, typically achieved with a high hydrogel content, promotes drug retention at the injection site (e.g., peritumoral areas), minimizing systemic exposure and off-target effects, and enhancing local targeting precision [79]. In contrast, rapid initial release—associated with a high EV content—increases the immediate concentration of free vesicles in circulation or interstitial fluids. While this may accelerate therapeutic onset, it also elevates the risk of hepatic clearance, phagocytic uptake, and premature dilution before reaching the target site—potentially compromising targeting efficiency unless the vesicles possess inherent targeting ligands.

Optimization of therapeutic outcomes depends on the specific treatment objective. For slow-growing tumors or conditions requiring sustained factor delivery (e.g., anti-angiogenic agents, immunomodulators), prolonged release kinetics enabled by a high hydrogel content is advantageous. Conversely, rapidly progressing tumors requiring prompt cytoreduction benefit from an initial burst release, typically achieved via high EV loading, though this necessitates careful control to limit systemic exposure [79].

Designing EV-loaded hydrogel systems is complicated by EV heterogeneity and dynamic *in vivo* factors—such as protein corona formation, enzymatic degradation, and interstitial fluid flow—which hinder the achievement of consistent and reproducible EV loading ratios across different applications and batches. To identify optimal loading ratios for various disease types, *in vitro* release profiling and *in vivo* assessments (e.g., bioimaging and biodistribution studies) remain essential evaluation methods. Integration of release kinetics models, such as Higuchi and Korsmeyer-Peppas, can facilitate the prediction of the release profile required to achieve therapeutic goals. If the final controlled-release performance is suboptimal, modifying hydrogel composition—such as by incorporating EV-binding ligands like heparin—may enhance drug loading capacity without compromising release controllability [80].

#### 4.2.2 Degree of Cross-Linking and Network Structure

The degree of cross-linking directly impacts the internal network structure of hydrogels, significantly influencing drug diffusion rates and tumor treatment efficacy. It reflects the density of chemical bonds between polymer chains, which in turn affects key properties such as pore size, connectivity, and mechanical strength [81,82]. Batta-Mpouma et al. used two cross-linking agents, glutaraldehyde and epichlorohydrin, at varying concentrations to produce nanoparticle-based hydrogels with tunable physicochemical properties. Their results showed that hydrogel strength, when cross-linked with glutaraldehyde and epichlorohydrin, was inversely related to the degree of cross-linking [83].

In high cross-linking hydrogels, the polymer chains are tightly wound and interconnected, forming a dense, well-organized network structure. For example, when high concentrations of glutaraldehyde are used in gelatin hydrogels, the internal pore size is significantly reduced, with most pores in the nanoscale range, resembling a fine “screen” [77]. This microstructure creates a tortuous diffusion path for drug molecules, significantly increasing diffusion resistance. This ultimately leads to a slower and more persistent release of dynamics. For small-molecule chemotherapeutic agents like cisplatin, high cross-linking hydrogels reduce the diffusion coefficient by more than an order of magnitude compared to low-crosslinking hydrogels, leading to a significantly slower drug release rate and sustained release characteristics. In tumor treatment, this property helps maintain stable drug concentrations at the tumor site, preventing fluctuations and continuously inhibiting tumor cell growth. This is particularly beneficial for slow-growing tumors that require prolonged drug intervention, gradually reducing tumor progression.

In contrast, hydrogels with a low degree of crosslinking have a loose, open network structure with large pores and high connectivity, resembling “channels” that facilitate fluid passage. In such a structure, drug molecules can move freely, significantly accelerating the diffusion rate [84]. Studies have shown that a reduced crosslinking density, achieved by lowering the amount of crosslinking agent, allows a large number of drug molecules to diffuse through the pores during the early stages of release. This results in a rapid increase in drug concentration at the tumor site, reaching a threshold necessary for effective tumor cell destruction [85]. This is particularly beneficial for fast-growing, aggressive tumors, as it helps limit tumor growth before widespread proliferation occurs. However, hydrogels with a low degree of crosslinking exhibit relatively poor *in vivo* stability [86]. In the complex microenvironment of blood circulation or tumor tissue, these hydrogels are prone to premature structural breakdown due to external forces, enzymatic hydrolysis, and other factors. This leads to early drug leakage, which hinders effective delivery to the tumor site and compromises both the precision and long-term efficacy of treatment. Therefore, when designing a drug delivery system consisting of hydrogels loaded with extracellular vesicles, the degree of crosslinking and network structure must be carefully optimized based on factors such as tumor type, treatment stage, and route of administration. This ensures the optimal balance of drug delivery and maximizes therapeutic benefits.

A high degree of crosslinking imparts excellent mechanical strength and resistance to destructive forces (e.g., tissue compression, fluid flow), enzymatic degradation (e.g., matrix metalloproteinases, MMPs), and hydrolytic cleavage. This improves the persistence of the drug reservoir and enhances the local retention of vesicles at the injection site, which is essential for achieving precise, site-specific targeting [87]. In contrast, low crosslinking density leads to accelerated structural degradation—via bulk or surface erosion—under physiological triggers such as mechanical stress or enzymatic hydrolysis. This premature drug release impairs localization, significantly compromising targeting accuracy and therapeutic efficacy.

The degree of crosslinking is inversely correlated with the equilibrium swelling ratio. Highly crosslinked hydrogels exhibit reduced swelling, limiting pore expansion during hydration and maintaining a smaller mesh size over time [83]. In contrast, lightly crosslinked gels swell extensively, enlarge pore size, and accelerate drug release.

In designing hydrogel-loaded EV systems, although increased crosslinking enhances mechanical stability and sustains release, it also reduces drug diffusion rates and may limit intratumoral cellular bioavailability. Highly crosslinked networks may also physically hinder the mobility of large EVs. Moreover, many effective chemical crosslinkers, such as glutaraldehyde, raise concerns regarding biocompatibility due to potential residual toxicity [88]. Excessive crosslinking or harsh loading conditions may compromise EV membrane integrity, surface marker expression, and overall biological activity [89].

To address these challenges, the degree of crosslinking can be fine-tuned by varying the type and concentration of crosslinking agents (e.g., glutaraldehyde, genipin, EDC/NHS) during hydrogel design [90]. Alternatively, enzyme-responsive or pH-sensitive hydrogels—such as those incorporating MMP-cleavable peptides—can be developed to achieve tumor microenvironment (TME)-triggered site-specific degradation [91]. Hybrid crosslinking strategies, combining physical (e.g., ionic or hydrogen bonding) and chemical methods, can improve stability while minimizing embrittlement and enabling stimuli-responsive disintegration [92].

Experimental evaluations included rheological testing (mechanical properties), swelling studies, scanning and atomic force microscopy (morphological analysis), and fluorescence recovery after photobleaching (FRAP) to assess diffusion and guide optimization of hydrogel–EV systems.

## 5 The Mechanism of the Drug Delivery System to Deliver Anti-Tumor Drugs

**Figure 7:**
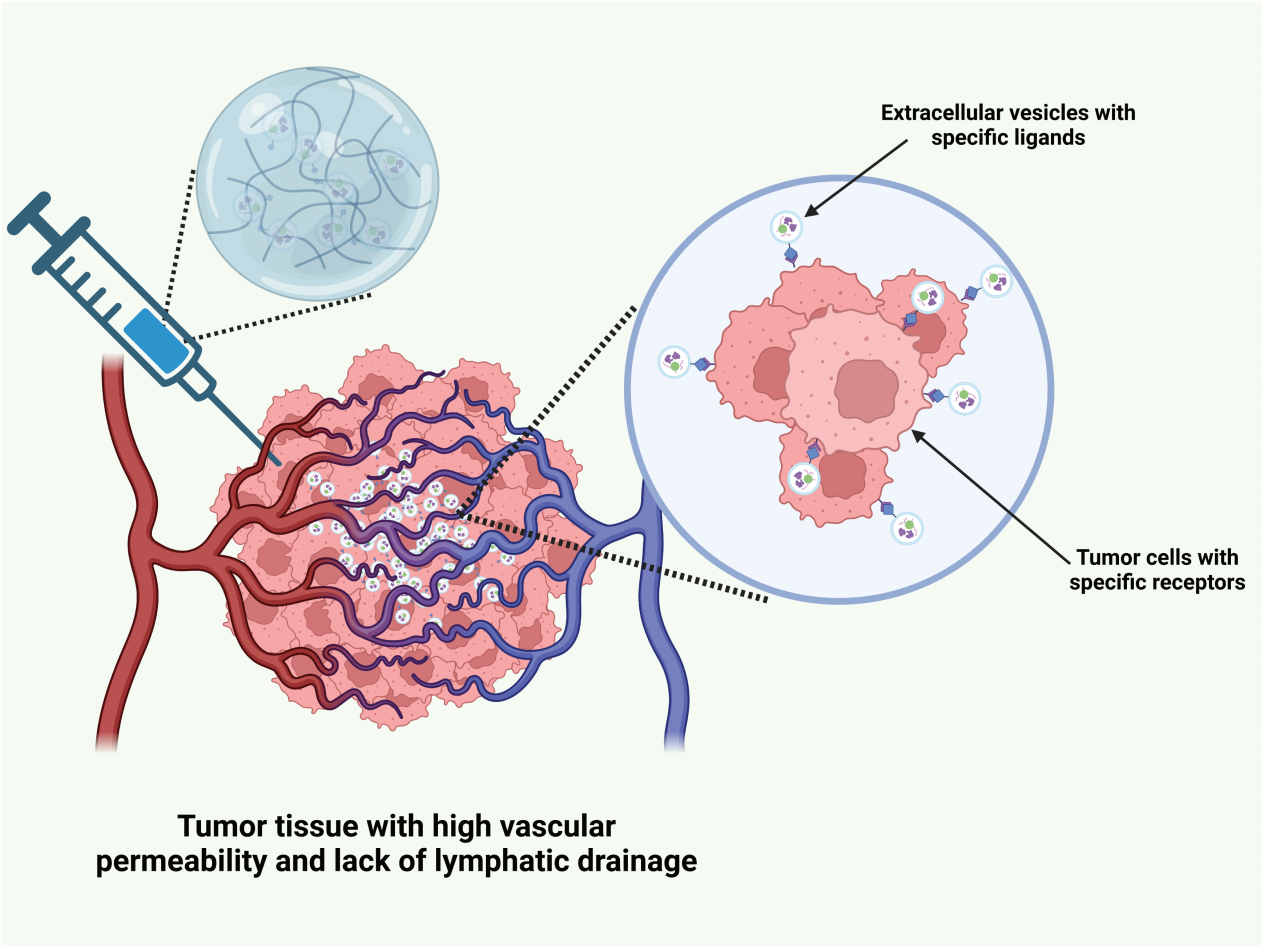
The mechanism of drug delivery system of hydrogel carrying extracellular vesicles for transporting anti-tumor drugs. This picture was drawn by Biorender. Agreement number: YS28Q5IWI2

Drug delivery systems utilize two primary mechanisms for anti-tumor drug delivery: passive targeting and active targeting. Passive targeting exploits the enhanced permeability and retention (EPR) effect of tumor tissues, enabling nanoscale drug delivery systems to penetrate and accumulate within tumor tissue, resulting in localized drug enrichment [93]. Active targeting facilitates precise drug delivery through the specific binding of ligands on extracellular vesicles to receptors on tumor cells, such as the interaction between folate and folate receptors, which induces endocytosis [94] (Fig. 7).

### 5.1 Passive Targeting Mechanism

#### 5.1.1 High Permeability and Retention Effect of Tumor Tissue

The physiological and pathological characteristics of tumor tissue naturally support the passive targeting of drug delivery systems consisting of hydrogels loaded with extracellular vesicles. During tumor progression, angiogenesis is triggered to meet the increased demand for nutrients and oxygen by rapidly proliferating cancer cells [95]. This process entails the upregulation of angiogenic factors, including vascular endothelial growth factor (VEGF), which stimulate endothelial cell proliferation, migration, and tube formation. In contrast to the regular, mature vascular network of normal tissues, tumor blood vessels are often highly disorganized [96]. The gap between vascular endothelial cells is significantly widened, ranging from 100 to 780 nm, far exceeding the tight junctions in normal endothelial cells (usually less than 2 nm), thereby greatly enhancing the permeability of the blood vessel wall. This increases the permeability, facilitating drug delivery systems to cross the vascular barrier and reach the tumor tissue.

Tumor tissue also lacks an effective lymphatic drainage system [97], hindering the removal of macromolecules and nanoparticles, which remain in the tumor site for extended periods [98]. Upon reaching the tumor via the bloodstream, the hydrogel-loaded cell membrane vesicle drug delivery system can penetrate the wide gaps in tumor blood vessel walls and enter the tumor stroma, owing to its nanoscale to micrometer size [99]. Once inside, the drug gradually accumulates in the tumor tissue due to poor lymphatic drainage, resulting in passive targeting and enhanced drug concentration at the tumor site. Compared to normal tissues, drug levels in the tumor can increase severalfold or even by orders of magnitude, significantly improving anti-tumor efficacy while minimizing toxicity and side effects in healthy tissues.

#### 5.1.2 Passive Targeting Dependent on Size

Size plays a critical role in the passive targeting of drug delivery systems. For instance, nanoscale delivery systems often demonstrate superior tumor penetration. When the particle size is less than 100 nm, these delivery systems exploit the loose extracellular matrix and disordered vascular structure of tumor tissue to penetrate deeper into the tumor. Initially, they can rapidly accumulate at the porous tumor periphery, inducing a swift killing effect on surrounding tumor cells. Smaller nanoparticles (e.g., 30 nm) are more flexible, allowing them to traverse the poorly permeable tumor interstitial space and diffuse rapidly throughout the tumor tissue. However, a disadvantage is that a rapid clearance rate may result in a short retention time at the tumor site. Relatively larger nanoparticles (e.g., 100–200 nm) exhibit a slightly slower diffusion rate, but they are more readily trapped by tumor blood vessels, resulting in longer retention times and sustained drug release within the tumor tissue [99].

### 5.2 Active Targeting Mechanism

#### 5.2.1 Ligand-Receptor Interactions on the Surface of Extracellular Vesicles

Extracellular vesicles are commonly functionalized with specific ligands that selectively bind to receptors on the surfaces of tumor cells. For instance, folate receptors are overexpressed on the surfaces of many tumor cells, including those in ovarian, breast, and lung cancers, making them a prime target for targeted therapy [100–102]. Extracellular vesicles can be engineered to display folate molecules on their surfaces through genetic modifications or chemical alterations. Upon entering the bloodstream, these drug-loaded vesicles specifically target tumor cells due to the strong affinity between folate and its receptors.

At the molecular level, folate binding to folate receptors initiates receptor-mediated endocytosis. Tumor cell surface folate receptors specifically recognize and bind folate, which induces cell membrane invagination and the formation of endosomes that encapsulate the vesicles, functioning as intracellular ‘warehouses’ for cargo delivery. As endocytosis proceeds, the endosome fuses with the lysosome. If the drug delivery system is engineered to be responsive to the acidic lysosomal environment, such as through chemical bonds that break in low pH conditions, the drug is precisely released within the lysosome, ensuring targeted delivery to the tumor cell’s interior [103]. This approach minimizes accidental damage to normal cells while significantly improving treatment efficacy and safety.

However, this therapeutic approach also presents several limitations. First, tumor cells display marked heterogeneity in receptor expression [104]. Within the same tumor lesion, certain cells may exhibit low receptor expression or complete loss of receptor function. This heterogeneity not only compromises the completeness of targeted drug delivery but may also facilitate the selective expansion of drug-resistant subclones, leading to therapeutic resistance. Second, while chemical modification of extracellular vesicles can enhance their pharmacokinetic properties, such modifications may alter the spatial conformation and stability of surface ligands. Moreover, exogenous modifications may activate immune recognition pathways, eliciting adverse responses such as complement activation or macrophage-mediated phagocytosis [105].

#### 5.2.2 Immune Cell-Mediated Targeting

Immune cells are central to the tumor immune response. When incorporated into hydrogel-based drug delivery systems loaded with extracellular vesicles, immune cells enhance targeted therapy and enable synergistic immunotherapy. For instance, the tumor microenvironment releases chemokines, such as macrophage colony-stimulating factor (M-CSF), which recruit macrophages to the tumor site. Exosomes from M1-polarized macrophages were isolated via ultracentrifugation and sonication to load paclitaxel, leveraging this characteristic [106].

The macrophage exosomes that ingested the drug were like “immune warriors” carrying “weapons”. With their natural tumor tendencies, they smoothly crossed the complex extracellular matrix barrier around the tumor tissue and accurately arrived near the tumor cells. Upon drug release, it directly affects tumor cells, disrupting key biological processes like proliferation, migration, and invasion. Additionally, immune regulatory factors released by activated macrophages, including tumor necrosis factor-α (TNF-α) and interleukin-12 (IL-12), further recruit and activate immune cells in the body. These include T lymphocytes and NK cells, which trigger a systemic anti-tumor immune response, creating a “ring of suppression” around tumor cells, significantly enhancing treatment efficacy and offering new hope for cancer therapy [107].

In the clinical translation of active tumor-targeted therapies, immune-related challenges have emerged as critical bottlenecks limiting therapeutic efficacy. Tumor cells promote macrophage polarization towards the tumor-supportive M2 phenotype by secreting factors such as TGF-β and PD-L1, which suppresses macrophage phagocytic activity and facilitates immune evasion [108]. Furthermore, extracellular vesicles are readily recognized by the reticuloendothelial system and rapidly phagocytosed by hepatosplenic macrophages via pattern recognition receptors, resulting in their immune clearance [109].

## 6 Application in Tumor Therapy

The hydrogel-loaded extracellular vesicles (EVs) delivery system has extensive applications in tumor treatment, including the delivery of chemotherapy drugs, immunotherapy drugs, combined therapy, and targeted therapy drugs (Table 4). Through synergistic effects, it enhances therapeutic efficacy and provides multiple effective strategies for tumor treatment.

**Table 4:** Comparison with different treatment methods

Treatment method	Subcategory	Main findings	Relative advantage	References
Chemotherapy drug delivery	Improve the stability and stability of chemotherapy drugs	The paclitaxel loading system prolongs the increased circulation time, increases. accumulation at the tumor site, and reduces the allergic and neurotoxicity of traditional solvents	Improve drug stability, reduce solvent-related side effects, and enhance tumor targeting	[112]
	Enhance the uptake efficiency of tumor cells	The doxorubicin-loaded CLEVs/GelMA system inhibits tumor migration in a triple-negative breast cancer model, while the gemcitabine-loaded system enhances glioblastoma cell uptake	Increase the intracellular concentration of drugs to overcome drug resistance	[16,116]
Delivery of immunotherapy drugs	Activate the anti-tumor immune response	The BCG-exosome-hydrogel system enhances the infiltration of immune cells at the tumor site and promotes the secretion of cytokines (IL-12, IFN-γ)	Precise targeted immune activation reduces immune side systemic effects and enhances the persistence of immune responses	[17,120]
	Overcome immune escape	The anti-PD-L1 exosome-hydrogel system reactivates T cell activity and inhibits tumor growth	Reverse the immunosuppression of the tumor microenvironmentand enhance the sensitivity of immunotherapy	[122,126]
Combined therapy	Chemotherapy-immune combination	The combined system is more effective than single therapy in reducing tumor volume, prolonging survival, and overcoming chemotherapy resistance	Synergistically enhance therapeutic effects and reduce dose-dependent toxicity of single therapy	[128,129]
	Multi-mode collaboration	The photothermal-chemotherapy combined system triggers hyperthermia and drug release through near-infrared light, and regulates tumor cell proliferation through gene-chemotherapy combination	Multi-target synergy can enhance the success rate of treatment, especially for refractory tumors	[131,132]
Targeted therapeutic drug delivery	Precise targeted delivery	The VH298-derived EVS-hydrogel system promotes diabetic wound healing through the HIF-1a/VEGFA pathway	Increase the local concentration of the drug. reduce damage to normal tissues, and adapt to the dynamic changes of the tumor microenvironment	[18,134]

### 6.1 Delivery of Chemotherapy Drugs

#### 6.1.1 Improving the Stability and Solubility of Chemotherapeutic Agents

Chemotherapeutic drugs, key “weapons” in tumor treatment, often face challenges with stability and solubility in clinical applications. Hydrogel-loaded cell membrane vesicle drug delivery systems offer an effective solution to these issues [110]. For instance, paclitaxel, a widely used chemotherapeutic agent, has poor water solubility. Common clinical cosolvents, such as Cremophor EL and absolute ethanol, are typically used in a 1:1 ratio [111]. However, these co-solvents are associated with adverse reactions, including allergic responses, neurotoxicity, and the formation of small particles in the bloodstream that encapsulate paclitaxel, hindering its therapeutic activity. The introduction of paclitaxel into hydrogel-loaded cell membrane vesicle delivery systems significantly reduces these issues. The three-dimensional network structure of the hydrogel creates a stable microenvironment for paclitaxel, where it is either physically embedded or chemically bonded, preventing premature degradation or precipitation from the bloodstream. Hydrophilic hydrogels, such as PEG, contain hydrophilic groups that form weak interactions with paclitaxel molecules, improving the drug’s dispersion and reducing agglomeration. Additionally, the bilayer structure of extracellular vesicles further protects paclitaxel from environmental variations.

Studies have shown that these EVs enhance the *in vivo* solubility of paclitaxel, enabling rapid release of therapeutic concentrations at the tumor site. Paclitaxel encapsulated in EVs demonstrates prolonged circulation time and increased accumulation at tumor sites compared to traditional formulations, thereby improving tumor treatment outcomes. This approach addresses the limitations of conventional paclitaxel formulations and opens the door to potential breakthroughs in clinical chemotherapy [112]. The hydrogel system offers a protective “shelter” for drug-loaded extracellular vesicles, improving drug stability, minimizing toxic side effects from vesicle rupture or premature drug release, and enabling intelligent responsiveness through customized functional designs [113].

#### 6.1.2 Enhance the Tumor Cell Uptake of Chemotherapeutic Drugs

In addition to improving the stability and solubility of chemotherapeutic drugs, hydrogel-loaded vesicles significantly enhance drug uptake by tumor cells, thereby boosting anti-tumor efficacy [114,115]. Tumor cell membranes feature specific receptors and transporters that can be targeted by EVs through surface modification with corresponding ligands, facilitating the internalization of the vesicles. Cui and colleagues employed GelMA hydrogel as a delivery platform, incorporating citrus lemon-derived extracellular vesicles (CLEVs) loaded with the chemotherapeutic drug doxorubicin (DOX). The GelMA hydrogel demonstrated outstanding biocompatibility along with adjustable mechanical characteristics. A high level of methacrylation increased the hydrogel’s stiffness, facilitating stable CLEVs encapsulation and prolonged DOX release. Additionally, CLEVs transported bioactive components that specifically targeted tumor cells, producing a synergistic effect alongside DOX. *In vivo* studies confirmed that this delivery system effectively suppressed tumor cell migration and aggregation, exhibiting significant therapeutic potential in a triple-negative breast cancer (TNBC) model [16]. Gazaille et al. developed hydrogels loaded with EVs carrying gemcitabine for the treatment of glioblastoma (GBM). The results demonstrated that this system accelerated the internalization of gemcitabine and enhanced its cytotoxic effects [116]. In drug-resistant tumor cells, the overexpression of drug transporters accelerates the efflux of chemotherapy agents, limiting their effective intracellular concentration [117]. The hydrogel-vesicle delivery system can circumvent this resistance by fusing with or being endocytosed by the tumor cells, directly delivering the chemotherapy drugs and preventing interaction with drug-resistant transporters on the cell membrane.

Studies have used doxorubicin as a model drug to compare the uptake efficiency of free doxorubicin and doxorubicin loaded in exosome-based delivery systems in tumor cells [117]. The results indicated that, at the same drug concentration and incubation time, the uptake of doxorubicin in the drug-loaded system was significantly higher than in the free drug system. This enhanced uptake is attributed to the specific binding of targeted ligands on extracellular vesicles to receptors on tumor cells [117]. Additionally, the responsive swelling or degradation of hydrogels in the tumor microenvironment brings the drug closer to the tumor cell surfaces, further enhancing drug uptake efficiency [118]. Efficient uptake of chemotherapeutic drugs by tumor cells not only improves the targeted action but also significantly increases cytotoxicity. This strategy holds promise in overcoming tumor drug resistance and is likely to enhance chemotherapy efficacy, ultimately improving patient survival rates.

### 6.2 Delivery of Immunotherapy Drugs

#### 6.2.1 Activation of Anti-Tumor Immune Responses

EVs-loaded hydrogel drug delivery systems offer unique advantages in tumor immunotherapy, providing innovative methods to activate anti-tumor immune responses. Immune adjuvants, which enhance immune responses to tumor-associated antigens, are crucial components of immunotherapy. However, traditional drug delivery methods often struggle to precisely target immune adjuvants to the tumor site, leading to systemic adverse immune reactions [119]. This challenge is effectively addressed by hydrogel-loaded cell membrane vesicle systems. For example, the classical immune adjuvant Bacillus Calmette-Guerin (BCG), when incorporated into a tumor cell-derived exosome-based delivery system combined with degradable hydrogels, allows for precise and sustained release at the tumor site. This system leverages the tumor-targeting capability of exosomes and the prolonged release properties of hydrogels [17].

Upon reaching the tumor site, EVs interact with antigen-presenting cells (APCs), such as dendritic cells (DCs), which then mature and migrate to the lymph nodes. There, they efficiently present tumor antigens to T lymphocytes, leading to the proliferation and activation of cytotoxic T lymphocytes (CTLs). Meanwhile, BCG is gradually released from the hydrogel, stimulating the secretion of cytokines, including IL-12 and IFN-γ, from immune cells. These cytokines enhance CTL-mediated cytotoxicity and recruit additional immune cells to the tumor site. Natural killer (NK) cells, macrophages, and other immune cells join the immune response, creating a robust attack on tumor cells and inhibiting tumor growth and metastasis. This delivery system significantly improves immune cell infiltration into tumor tissues, marking a significant advance in tumor immunotherapy. It is expected to become an ideal carrier for immunotherapeutic agents, potentially overcoming tumor immune escape mechanisms [120].

#### 6.2.2 Overcoming Immune Escape

Immune evasion mechanisms employed by tumor cells pose a significant challenge to delivery systems that use hydrogels loaded with extracellular vesicles provide an innovative solution to these cells often evade immune recognition and attack by downregulating major histocompatibility complex (MHC) molecules and upregulating immune checkpoint molecules, such as programmed death ligand 1 (PD-L1) [121]. Hydrogel-loaded extracellular vesicles can intervene at multiple levels, restoring the body’s immune surveillance function.

On one hand, extracellular vesicles can be genetically engineered to carry molecules or nucleic acid sequences that upregulate MHC molecule expression in tumor cells [122,123]. Daza Zapata and her team altered the fatty acid profiles of extracellular vesicles by employing Janus kinase (JAK) inhibitors, thereby granting the vesicles the capability to regulate immune responses [124]. On the other hand, extracellular vesicles carrying immune checkpoint inhibitors can be incorporated into hydrogels for local drug delivery, targeting the immunosuppressive pathways mediated by these molecules. For example, when anti-PD-L1 antibodies are loaded onto exosomes and combined with hydrogel, the microvesicles exploit the natural tumor tropism of macrophages to precisely target tumor cells upon delivery to the tumor tissue. At the tumor microenvironment’s temperature, the hydrogel undergoes a phase transition, releasing the anti-PD-L1 blocks the PD-1/PD-L1 interaction between tumor cells and T lymphocytes, thereby reactivating T lymphocyte-mediated anti-tumor activity. It breaks tumor immune tolerance, creates favorable conditions for immunotherapy, and advances tumor immunotherapy towards greater efficiency and precision [125,126].

### 6.3 Combined Treatment Application

#### 6.3.1 Chemotherapy-Immunotherapy Combination Therapy

The cell membrane vesicle-loaded hydrogel drug delivery system holds significant potential for combining chemotherapy and immunotherapy, providing a novel approach to tumor treatment. This system precisely delivers both chemotherapy and immunotherapy agents to the tumor site, resulting in a synergistic effect that significantly improves therapeutic drug’s target and kills tumor cells, induces immunogenic cell death, and releases tumor-associated antigens, which activate the immune system. Immunotherapy enhances the activity of immune cells, such as cytotoxic T lymphocytes (CTLs) and natural killer (NK) cells, improving their ability to recognize and eliminate tumor cells [127].

When chemotherapeutic and immunotherapeutic drugs are combined, they are encapsulated in a delivery system composed of exosomes and hydrogels [127]. Upon reaching tumor tissue, chemotherapeutic drugs exert rapid cytotoxic effects, inducing apoptosis in tumor cells and releasing abundant tumor antigens [128] Simultaneously, immunotherapeutic agents are gradually released as the hydrogel degrades, thereby activating surrounding immune cells and promoting their proliferation and activation. Upon recognition of tumor antigens, immune cells initiate a strong immune response, attacking and suppressing tumor cells [129]. Studies have shown that compared to chemotherapy or immunotherapy alone, combination therapy more effectively inhibits tumor growth, reduces tumor volume, overcomes chemotherapy resistance, and prolongs patient survival. This approach offers new hope for patients with advanced tumors and is poised to become a leading strategy in future cancer therapies [130].

#### 6.3.2 Multi-Mode Combination Therapy

In addition to combined chemotherapy-immunotherapy, the drug delivery systems consisting of hydrogels loaded with extracellular vesicles enable multimodal combination therapy, further extending the possibilities for cancer treatment [131]. By integrating photothermal therapy, gene therapy, and other advanced treatment modalities, this system maximizes the advantages of each method, achieving complementary effects and improving tumor treatment outcomes.

In combined photothermal therapy and chemotherapy, researchers have integrated nanomaterials with photothermal conversion properties (e.g., gold nanorods, carbon nanotubes) with chemotherapeutic drugs [131]. These photothermal conversion nanomaterials combined with chemotherapeutic drugs can also be encapsulated within the hydrogel and cell membrane vesicle system. Upon reaching the tumor site, the system is exposed to external near-infrared light, enabling the nanomaterials to rapidly convert the light into heat, thereby elevating the local temperature of the tumor tissue. Thermal stimulation also promotes hydrogel swelling or degradation, accelerating chemotherapeutic drug release and enhancing tumor cell cytotoxicity. Meanwhile, the targeting properties of extracellular vesicles ensure precise drug delivery to tumor tissues, minimizing damage to normal tissues.

In gene therapy combined with chemotherapy, plasmids or nucleic acid sequences carrying specific therapeutic genes (e.g., tumor suppressor genes, immunomodulatory genes) are loaded into the system to synergize with chemotherapy drugs [132]. While killing tumor cells, chemotherapeutic drugs enhance conditions for gene therapy, such as improving nucleic acid uptake by tumor cells. Therapeutic genes regulate tumor cell behavior at the genetic level, inhibiting cell proliferation, inducing apoptosis, and enhancing the body’s anti-tumor immune response. Through a multi-dimensional, multi-target approach, therapeutic genes efficiently treat tumors, offering new hope for patients and advancing tumor therapy towards precision and diversification [133].

### 6.4 Delivery of Targeted Therapeutic Drugs

In oncology, hydrogel-based delivery platforms for EVs have yet to attract adequate research attention. Current studies mainly concentrate on areas such as promoting diabetic wound healing, alleviating intervertebral disc degeneration, and other related conditions. For instance, hydrogels containing VH298-derived EVs have been utilized for treating diabetic wounds. Experimental findings demonstrated that the bioactive components within EVs stimulate cell proliferation and migration at the wound site, enhance angiogenesis by activating the HIF-1α/VEGFA signaling pathway, and that hydrogels provide a stable microenvironment, thereby extending the retention time and biological activity of EVs at the injury site. The synergistic interaction between EVs and hydrogels markedly accelerates the healing process of diabetic wounds [18]. To tackle intervertebral disc degeneration, some researchers incorporated EVs derived from mesenchymal stem cells (MSCs), which contained Glutaredoxin3 (GLRX3), into hydrogels and subsequently injected them into animal models of disc degeneration. The results showed that the hydrogels enabled the gradual release of EVs, which then released GLRX3, boosting cellular antioxidant defenses. This process helped prevent the buildup of reactive oxygen species (ROS) and the subsequent progression of cellular aging *in vitro* [134].

Although still underexplored in oncology, this delivery system offers substantial promise for targeted tumor therapy. Tumor cells exhibit unique biological features and microenvironments, which can be leveraged by hydrogel-loaded extracellular vesicle (EV) delivery systems. On one hand, EVs can enhance targeting precision through gene-editing, effectively directing drugs to tumor cells, increasing local drug concentrations, and improving therapeutic outcomes while minimizing damage to healthy tissues. On the other hand, the combined protective and adaptable characteristics of EVs and hydrogels reduce drug consumption, allow for adaptation to complex *in vivo* conditions, and facilitate smart drug release in response to changes in the tumor microenvironment (such as pH, temperature, and enzyme levels), thereby optimizing cancer treatment [14].

## 7 Summary and Prospect

The drug delivery systems consisting of hydrogels loaded with extracellular vesicles are an innovative strategy with significant potential in cancer treatment and tissue regeneration. Hydrogels, with their excellent biocompatibility, controllable swelling, and drug release properties, create a stable microenvironment for drugs and extracellular vesicles, protecting them from *in vivo* environmental degradation. Extracellular vesicles leverage their unique targeting ability and immunomodulatory function to precisely deliver drugs to the tumor site, enhancing antitumor efficacy and activating the immune response, offering a potential solution to tumor immune escape. The combination of these two systems utilizes passive and active targeting mechanisms to efficiently deliver various anticancer drugs, including chemotherapy and immunotherapy agents. This synergistic effect significantly inhibits tumor growth, metastasis, and prolongs patient survival.

However, significant challenges remain on the path to clinical application. During large-scale production, technical challenges such as uniform mixing and precise cross-linking control arise. The lack of a standardized quality control system compromises the stability and consistency of the product across batches. Factors such as complex enzymatic hydrolysis, metabolic variations, and individual differences present significant risks to the stability of the delivery system and the precision of drug release. These factors can lead to adverse outcomes such as premature drug leakage and unexpected release, compromising therapeutic efficacy and safety.

### 7.1 Facing the Challenge

#### 7.1.1 Scale Preparation and Quality Control

Large-scale preparation and quality control are key challenges on the path to clinical application. Most existing preparation methods remain at the laboratory scale, making them inadequate for meeting the clinical demands of batch production. While the physical blending method is simple, it is challenging to ensure the even dispersion of hydrogels and extracellular vesicles and prevent agglomeration during large-scale mixing. The chemical cross-linking method involves complex reaction conditions, including cross-linking agent concentration, temperature, time, and other parameters. As production scale increases, accurately controlling these conditions becomes increasingly difficult.

Establishing comprehensive and reliable quality standards and detection methods for quality control is challenging due to the complexity of the delivery system, which encompasses multiple factors, such as the physicochemical properties of hydrogels, the biological activity of extracellular vesicles, and the stability of their integration. Currently, no unified standard exists for detecting the purity and activity of extracellular vesicles. Different laboratories use varying methods, resulting in non-comparable results. Ensuring stability across batches, including swelling performance, degradation rate, and other hydrogel indicators, is challenging. This poses potential risks for clinical application, leading to unpredictable treatment outcomes and possible adverse reactions.

Meanwhile, the high production costs of raw materials significantly impede clinical translation. Natural polymers commonly employed in hydrogels, such as hyaluronic acid, as well as synthetic materials, tend to be costly. Additionally, isolating extracellular vesicles (EVs) from cell cultures requires expensive culture media, sophisticated equipment, and prolonged cell expansion. For example, conventional cell culture demands stringent control of parameters such as temperature and humidity. Moreover, EV yield increases nonlinearly with cell density and culture duration, further escalating unit production costs.

#### 7.1.2 The Influence of Complex Environment In Vivo

Upon entering the body, the drug delivery system encounters a complex and variable physiological environment, where various factors affect its stability and drug release. The body’s enzymatic system can degrade foreign substances to protect against pathogens. However, the hydrogel delivery system carrying extracellular vesicles may also be recognized as foreign material and targeted for degradation.

Changes in biomolecule and ion concentrations in the blood, along with variations in the microenvironment of tissues and organs—such as pH, osmotic pressure, and temperature—can significantly affect the physicochemical properties of the delivery system. In organs like the liver and kidney, high concentrations of local metabolites may disrupt the balance of hydrogel swelling, causing uncontrolled drug release. Tumor tissue’s unique metabolic characteristics, such as acidic conditions and low oxygen, can be leveraged for intelligent controlled release. However, if the delivery system’s response sensitivity is insufficient, precise and efficient drug release at the tumor site may not be achieved, and unintended drug release in normal tissues could cause side effects, increasing patient suffering and treatment risks.

### 7.2 Future Prospects

*Exploration of New Materials and Technologies*

The integration of life sciences and materials science is expected to drive a series of innovative breakthroughs in the hydrogel-loaded cell membrane vesicle drug delivery system. The rapid advancement of gene-editing technologies presents new opportunities for optimizing extracellular vesicles. Cutting-edge gene-editing techniques, such as CRISPR-Cas9, enable precise modifications of the gene sequence in extracellular vesicles, allowing for the regulation of surface protein expression and function. For example, introducing a tumor-specific target protein gene into vesicle-derived cells results in high expression of the protein on the surface of secreted vesicles, improving tumor cell recognition and binding, thus enhancing the precision of targeted drug delivery [135]. Simultaneously, knocking out genes related to immunogenicity or drug leakage on the vesicle surface can enhance vesicle stability and safety *in vivo*, providing a solid foundation for clinical application.

The field of hydrogels shows great promise, with ongoing research and development of smart hydrogels. Researchers are focused on designing and synthesizing smart hydrogels that respond to various *in vivo* microenvironmental signals, including temperature, pH, enzyme concentration, and biomolecule concentration. For tumor treatment, an intelligent hydrogel has been developed that simultaneously senses the acidic tumor microenvironment, highly expressed enzymes, and local temperature changes. Upon delivery to the tumor site, the hydrogel rapidly and precisely initiates the drug release process under the synergistic influence of multiple stimuli, ensuring efficient drug release at the tumor site and maximizing therapeutic efficacy [136]. Furthermore, nanocomposite hydrogels with unique physical and chemical properties are being developed by integrating nanotechnology with hydrogels. For instance, gold nanoparticles are incorporated into hydrogels to impart photothermal conversion properties. When irradiated with near-infrared light, nanocomposite hydrogels not only kill tumor cells via photothermal effects but also promote drug release, facilitating the synergistic effect of photothermal therapy and chemotherapy, offering a new approach to overcoming tumor challenges [137].

## 8 Summary

This review comprehensively summarizes recent advances in hydrogel-based extracellular vesicle (EV) drug delivery systems for tumor therapy, highlighting their synergistic potential to overcome limitations inherent to conventional treatments. Hydrogels, characterized by biocompatible three-dimensional networks and stimulus-responsive release profiles, protect EVs from degradation and facilitate controlled delivery. EVs inherently exhibit tumor-homing capabilities and immunomodulatory functions, enhancing targeting specificity and therapeutic efficacy. This delivery platform presents a promising strategy to overcome drug resistance and immune evasion during tumor treatment. Nevertheless, challenges—including scalable production, stringent quality control, and *in vivo* stability—must be addressed to facilitate clinical translation.

## Data Availability

All the data is available in the manuscript and there is no associated data.
